# Michigan State Board of Health

**Published:** 1888-03

**Authors:** 


### ABSTRACTS AND EXTRACTS.

### MICHIGAN STATE BOARD OF HEALTH.

The regular quarterly meeting was held in Lansing, January 10, 1888. The members present were Hon. John Avery, M. D., President; Professor Victor C. Vaughan, M D ; Arthur Hazlewood, M.D.; J. H. Kellogg, M. D.; and Henry B. Baker, M. D., Secretary.

MICHIGAN STATE LABORATORY OF HYGIENE.

Professor VICTOR C. VAUGHAN, M. D., made his first Quarterly Report of the Michigan State Laboratory of Hygiene, of which Professor Vaughan is Director. This complete report is to be published in the Annual Report of the State Board of Health for 1887.

Professor Vaughan's report includes three subjects:

(1) The important results of the investigations into the causation of typhoid fever, stating the details of the experiments whereby the "germs"—the bacilli of typhoid fever—were proved to be in the water supposed to have caused the typhoid fever at Iron Mountain, Michigan, in October, 1887; and whereby, through the injection of the "germs," a disease in some respects similar to typhoid fever was produced in an animal, and, through injection of a ptomaine formed by the germs and chemically separated from the germs, an abnormal rise of body-temperature was produced in an animal.

(2) The complete account of the four cases (three fatal) of tyrotoxicon poisoning near Milan, Michigan, in September, 1887, and the experiments indicating that the poison may be generated in soil saturated with decomposing milk.

(3) The investigations which exposed a fraud which was putting into the hands of pharmacists and physicians a drug claimed to be a harmless product of the honey-locust tree, but which was found to be a dangerous mixture of cocaine and atropine.

The Secretary of the Board was instructed to send circulars and blanks to the health officers of all cities and villages in Michigan urging a sanitary survey, a house-to-house inspection, next spring, and asking the health officers to mention the subject in their annual reports to the common councils or other local boards of health.

DIFFICULTIES IN THE RESTRICTION OF COMMUNICABLE DISEASES.

In efforts for restricting communicable diseases, the Secretary read a letter which had been received during the quarter, saying that considerable trouble is experienced in preventing the spread of diphtheria, from the fact that some physicians will not report cases that come under their care, and claim that there is no diphtheria, whilst other physicians pronounce simliar cases to be true diphtheria.

In response to the communication, a letter was sent from the office of the Board, stating that the safest way would be to consider every case of sore throat, in a locality where diphtheria prevailed, as suspected diphtheria, and to distribute pamphlets (issued by the State Board of Health) on the restriction of this disease to all the neighbors of those sick. In that way that class of physicians who do not regard the public health will not be depended upon to control public opinion, but after a time will be controlled by public opinion.

The local board of health in this locality in which diphtheria so prevailed was advised to meet and pass a resolution, which should stand as one of the "regulations" of the board, that in all cases of inflamed throat the local board of health, acting on the advice of the State Board, would require physicians and householders to report said cases to the health officer, in order that precautions might be taken, as in cases known to be diphtheria.

It was pointed out that, while diphtheria is, as a rule, *fatal to children* only, it is *spread* frequently by cases in *adults*, because they are not sufficiently well marked to make ordinary people believe they are diphtheria.

In all cases of sore throat precautions should be taken.

The following resolutions were adopted by the Board at that meeting:

Whereas, it is often difficult to recognize mild cases of diphtheria, or to distinguish such cases from a simple tonsilitis, pharyngitis, or larnyngitis, and

Whereas, such mild cases of diphtheria often communicate a dangerous and fatal form of diphtheria:

*Resolved,* That it is the duty of physicians and householders in reporting diseases dangerous to the public health, and of local health authorities in their efforts to restrict such diseases, in every case, to give to the public safety the benefit of the doubt, and in localities where diphtheria exists to regard cases of acute sore throat as suspected cases of diphtheria.

*Resolved,* That suspected cases of dangerous diseases should be reported, and precautionary measures should be taken.

INFLUENCE OF DRESS ON RESPIRATION.

Dr. J. H. Kellogg presented facts and memoranda of a paper, entitled, "The So-called 'Female Type of Respiration' the Result of Improper Dress, as Shown by the Study of Respiration in Indian and Chinese Women."

This paper embodies the results of the study of respiration in nineteen Chinese women, fifteen Indian women, and more than one hundred civilized women, by the aid of the pneumograph. The results show:

*First.* That there is but one normal type of respiration in human beings, contrary to the views held by our leading physiologists, who assert that normal respiration in adult women is costal, while in males it is of the abdominal type.

*Second.* That the so-called "male," or abdominal type of respiration, is the normal type for both men and women when the movements of the chest and abdomen are not restricted by improper dress.

Dr. Kellogg was requested to complete the paper for the annual report of the Board.

THE DRY-CLOSET SYSTEM OF DISPOSAL OF EXCRETA.

The committee appointed for the purpose of investigating the Smead System of Dry Closets as used in connection with public schools, reported that it had visited several school buildings and carefully examined the system of dry closets in use in them. In all but one building the working of the system appeared to be very satisfactory, the outside temperature being such as to insure good draft in any heated building provided with an efficient system of ventilation. In one building the odors present in the basement were, when it was first entered, exceedingly foul, the odors coming chiefly from the urinal. It was necessary to open the basement windows to clear the room, even in part, of the odors present. The fæcal matter of the vaults was, in two of the buildings visited, nearly dry; in the building referred to above it was in a highly putrescent condition.

The committee also visited a school building in Detroit in which this system had been recently introduced, but had not been long enough in use to enable us to judge of its efficiency. The conclusions reached by the committee were as follows:

1. The dry-closet system, as introduced in connection with the Smead-Rutten system of ventilation, with a sufficiently strong and constant draft, presents features of economy and conveniences which render it worthy of investigation.

2. The apparent success of the system is wholly due to the efficiency of the system of ventilation in connection with which it is introduced.

3. The system presents several features, however, which do not commend it to sanitarians, and which certainly suggest a further study and observation of the system before it can receive scientific indorsement.

*a*. There is always a possibility, and under some circumstances, as during the prevalence of adverse winds and in hot weather, a probability, of an interference with the ventilating system, by which fæcal odors may be carried, by a back-draft in the foul-air ducts, into the school rooms.

*b*. The system does not provide a satisfactory method of caring for the boys' urinal.

*c*. In the light of the most recent researches, the drying of fæcal matters, which is supposed to occur in the dry vault, does not destroy the germs of disease which may be contained in them; and the scattering of these germs, through their discharge into the open air, may be conducive to the wide dispersion of the infectious elements of diphtheria, typhoid fever, and, possibly, other grave maladies. This danger would, of course, be greatly aggravated in times of epidemics of any of these diseases.

4. On the whole we are obliged to express the opinion that the dry-closet system is not in the line of the best sanitary progress.

The committee wished especially to call attention to the fact that their inquiries thus far have been prosecuted at a season of the year when this system would necessarily appear at its best, and when its dangers and disadvantages are least likely to appear; and on this account they desired to withhold their final and complete report upon the dry-closet system until they shall have had opportunity to observe its working during the warm season of the year.

### DANGERS IN GASOLINE.

Dr. J. H. Kellogg, who had been appointed a committee to investigate the dangers in gasoline, made a report, embodying facts which he had collected, and including the views of leading insurance agents, etc., concerning the dangers in the use and storing of gasoline, and giving rules to be observed in handling this substance, declared to be "more dangerous than gunpowder." The report is to be published in the annual report of the State Board of Health for 1887.

### CONTAGIOUS DISEASES.

Compared with the preceding quarter (July, August, and September), reports received from all sources show the number of places at which diphtheria was reported to have increased by an average of eight places per month, scarlet fever to have increased by an average of nine places per month, typhoid fever to have decreased by an average of two places per month, and measles to have increased by an average of one place per month. One case of smallpox was reported in each quarter.

METEOROLOGY, AND SICKNESS FROM ALL CAUSES, COMPARED WITH THE PRECEDING QUARTER.

A comparison of the meterological conditions of the fourth quarter of 1887, with the meteorological conditions of the third quarter, shows the temperature to have been much lower, the absolute humidity and the day ozone less, the relative humidity more and the night ozone slightly more in the fourth quarter of 1887.

Compared with the preceding quarter (July, August, and September), the reports received from regular observers show a marked increase of pneumonia, bronchitis, influenza, tonsilitis, scarlet fever, diphtheria, rheumatism, and consumption of the lungs, and a marked decrease of diarrhœa, cholera morbus, dysentery, cholera infantum, intermittent fever, and whooping-cough in the fourth quarter of 1887.

